# Lung transplantation for late-onset non-infectious chronic pulmonary complications of allogenic hematopoietic stem cell transplant

**DOI:** 10.1186/s12931-021-01699-8

**Published:** 2021-04-07

**Authors:** Peter Riddell, Ram Vasudevan-Nampoothiri, Jin Ma, Lianne G. Singer, Jeff H. Lipton, Stephen C. Juvet

**Affiliations:** 1 Ajmera Transplant Centre and Toronto Lung Transplant Program, Toronto General Hospital, Toronto, Canada; 2 Hans Messner Allogenic Blood and Marrow Transplant Program, Princess Margaret Cancer Centre, Toronto, Canada; 3 Biostatistics Research Unit, University Health Network, University of Toronto, Toronto, Canada

**Keywords:** Allogenic hematopoietic stem cell transplantation, Lung transplantation, Infection

## Abstract

**Background:**

Late onset non-infectious pulmonary complications (LONIPCs) following allogenic hematopoietic stem cell transplantation (allo-HSCT) confer a significant mortality risk. Lung transplantation (LTx) has the potential to provide survival benefit but the impact of prior allo-HSCT on post-LTx outcomes is not well studied.

**Methods:**

This retrospective, single-centre cohort study assessed the post-LTx outcomes of adults with LONIPCs of allo-HSCT. Outcomes of LTx for LONIPCs were compared to propensity-score matched LTx controls (n = 38, non-HSCT) and recipients of re-LTx (n = 70) for chronic lung allograft dysfunction (CLAD).

**Results:**

Nineteen patients underwent DLTx for LONIPCs of allo-HSCT between 2003 and 2019. Post-LTx survival was 50% at 5-years. Survival to 1-year post-LTx was similar to matched controls (p = 0.473). Survival, conditional on 1-year survival, was lower in the allo-HSCT cohort (p = 0.034). An increased risk of death due to infection was identified in the allo-HSCT cohort compared to matched controls (p = 0.003). Compared to re-LTx recipients, the allo-HSCT cohort had superior survival to 1-year post-LTx (p = 0.034) but conditional 1-year survival was similar (p = 0.145).

**Conclusion:**

This study identifies an increased risk of post-LTx mortality in recipients with previous allo-HSCT, associated with infection. It supports the hypothesis that allo-HSCT LTx recipients are relatively more immunosuppressed than patients undergoing LTx for other indications. Optimisation of post-LTx immunosuppressive and antimicrobial strategies to account for this finding should be considered.

**Supplementary Information:**

The online version contains supplementary material available at 10.1186/s12931-021-01699-8.

## Introduction

Late onset non-infectious pulmonary complications (LONIPCs) allogenic haematopoietic stem cell transplantation (allo-HSCT) are a significant cause of morbidity and mortality [1]. These complications cover a wide spectrum of respiratory disease, but most commonly present as bronchiolitis obliterans or interstitial lung disease [2]—the aetiology of which is thought to include chronic graft-versus-host-disease (GVHD), toxicity of HSCT conditioning regimens and infection [3, 4].

The management of LONIPCs depends on diagnosis, and response to treatment is variable. Chronic pulmonary GVHD is often managed with immunosuppression, inhaled corticosteroids, and macrolide antibiotics. However, treatment response is frequently poor [5] and 5-year survival from diagnosis is often less than 50% [6, 7]. For other LONIPCs, such as pleuro-parenchymal fibroelastosis (PPFE) no effective medical therapy is known and progressive disease is common [8].

Lung transplantation (LTx) has the potential to provide both survival and quality of life benefits to patients who develop LONIPCs following allo-HSCT. However, as this is a rare indication for LTx, comprehensive evaluation of post-LTx outcome is lacking. Recipient co-morbidity associated with previous allo-HSCT is likely to impact the post-LTx risk profile, and the clarification of this risk is important to optimise both recipient selection and post-LTx management.

In this study, we describe our institutional experience of LTx for LONIPC of allo-HSCT. We compare post-LTx outcomes to propensity-score matched LTx controls and to recipients of re-LTx for chronic lung allograft dysfunction (CLAD). Our aim in selecting these control groups was to assess whether the presence of a prior allo-HSCT increases recipient risk relative to different LTx populations.

## Patients and methods

We retrospectively assessed the long-term outcomes of adults who underwent LTx for LONIPC of allo-HSCT. The study design was a matched-cohort study, with propensity-score matching performed to identify a non-HSCT LTx control group (2:1; n = 38). Patient demographics and post-transplant outcomes were obtained from the Toronto Lung Transplant database and confirmed by electronic healthcare records. The date of data censure was 31st December 2019.

### Inclusion and exclusion criteria

This study included adults who underwent double lung transplantation (DLTx) at the Toronto General Hospital between Jan 2003 and Dec 2019. Cystic fibrosis patients colonised with *Burkholderia cenocepacia* were excluded from the propensity-score matching process, as this pathogen has been associated with markedly impaired post-LTx survival [9]. Patients who underwent LTx following non-allogenic HSCT were also excluded. This study was performed with approval of the institutional review board at the University Health Network, Toronto.

### Post lung transplant immunosuppression

Induction immunosuppression was not routinely administered. Basiliximab IV 20 mg (day 0 and day 4) was administered if the initiation of calcineurin inhibitor was delayed. All patients received methylprednisolone IV 500 mg at the time of surgery, followed by 0.5 mg/kg 12 hourly for 3 doses. Maintenance immunosuppression included cyclosporine bid (based on trough levels), azathioprine 1.5-2 mg/kg od and prednisone 0.25 mg/kg od (tapering to 0.15 mg/kg over 3 months). Mycophenolate (Myfortic 720 mg bid or Cellcept 1 g bid) was substituted for azathioprine if the HLA screen was cPRA positive. Immunological risk at the time of lung transplant was classified as either standard or high risk, based on virtual or flow antibody cross match (CXM) results. Peri-operative antibody desensitisation was performed for patients with high immunological risk. This included 5 cycles of plasmapheresis, 1 g/kg immunoglobulin and anti-thymocyte globulin (ATG; 3 mg/kg if virtual CXM positive alone, or 5 mg/kg if flow CXM positive) [10]. Post-LTx immunosuppression strategy did not differ in patients undergoing LTx for cGVHD compared to this standard approach.

### Post lung transplant bronchoscopy

Surveillance bronchoscopy [including bronchoalveolar lavage (BAL) and transbronchial biopsy (TBBx)] occurred at 2 and 6 weeks, then at 3-, 6-, 9-, 12-, 18- and 24-months post-LTx. Additional monitoring occurred as per clinical need. Organisms identified from BAL were classified by pathogenic significance (Additional file 1: Table S1) [11]. BAL diagnostic tests included culture-based techniques (bacterial, fungal, and mycobacterial), viral assessment by polymerase-chain reaction (PCR) and galactomannan immunoassay. Category 1 pathogens were felt to represent clinically significant pathogens (e.g., Pseudomonas, Influenza) and category 2, usually significant pathogens (e.g., Aspergillus, *M. avium* complex). Other microorganisms were classified as usually insignificant (category 3) or insignificant (category 4).

### CMV monitoring, prophylaxis, and treatment

CMV prophylaxis, ganciclovir 5 mg/kg IV od, was commenced at the time of lung transplant. This was changed to valganciclovir 900 mg od once enteral medication could be tolerated. CMV prophylaxis continued for 9 months in CMV IgG donor positive/recipient negative cases (CMV D+/R−), 6 months in CMV IgG D+/R+ cases, and 6 months in CMV IgG D−/R+ cases. CMV IgG D−/R− cases did not receive CMV prophylaxis. Following the discontinuation of prophylaxis, serum CMV PCR was monitored weekly for 3 months. If valganciclovir or ganciclovir was withheld during the planned period of prophylaxis, serum CMV PCR was monitored weekly until recommenced. Further CMV PCR testing was performed as per clinical indication.

In the setting of CMV viraemia, asymptomatic patients were managed with valganciclovir 900 mg bid as an out-patient. The CMV PCR thresholds for treatment of asymptomatic patients were ≥ 500 IU/ml (CMV D+/R−) and ≥ 1000 IU/ml (CMV D+/R+ and CMV D−/R+). Symptomatic CMV infection was treated with IV ganciclovir 5 mg/kg od for a minimum of 2 weeks, or until CMV PCR was down trending and symptoms improving. At this time, ganciclovir was switched to valganciclovir 900 mg bid. Serum CMV PCR was monitored weekly, and treatment discontinued once two negative CMV PCR readings were achieved.

### Statistical analysis

The selection of a control group for this retrospective study was performed with propensity score matching, using the optimal match method [12]. The optimal match method produces matches that attain the smallest average propensity score distances across all matched pairs by utilising network flow optimization. The variables included in propensity score matching included recipient age, waiting list status at LTx admission (Additional file 1: Table S2), donor type (donation after cardiac or brain death), donor-recipient gender match, donor-recipient CMV match, immunosuppressive risk profile and follow-up time from LTx.

Data in the results section are expressed as mean (± standard deviation), median (interquartile range) or percentage. Continuous variables were compared using unpaired t-tests. Nominal variables were compared using Fisher’s exact test. Post-transplant survival, time to CLAD and freedom from de novo DSA (donor specific antibody) were estimated by Kaplan–Meier analysis. Hazard ratios for survival were assessed using the Mantel–Haenszel method. Cause-specific hazard ratios for risk of infection was performed using the Andersen-Grill model. The generalized estimating equations model, with an exchangeable correlation structure, was used to assess the association between allo-HSCT and severe CMV infections. Assessment of cumulative incidence functions for competing risk events were performed using Gray’s K-sample test. Statistical analysis was performed using GraphPad Prism Version 8.4.3 and R version 3.6.0.

## Results

### Patient characteristics

During the study period, nineteen patients underwent DLTx for LONIPCs of allo-HSCT. Chronic myeloid leukaemia (CML; n = 6) was the most common indication for allo-HSCT. 84.2% of patients received matched-related stem cell donations (Table 1). At the time of LTx referral, all patients had developed chronic GVHD affecting at least 1 organ site. Extra-pulmonary chronic GVHD was present in 15 patients, with skin being the most affected site (63.2%, n = 12). Extra-pulmonary GVHD was adequately controlled in all patients prior to LTx, with prednisone (100%; n = 19) and mycophenolate (52.6%; n = 10) being the most prescribed medications.

**Table 1:** Recipient characteristics at the time of allogeneic hematopoietic stem cell transplantation

	Allogenic HSCT Recipients (n = 19)
Age at HSCT, years	
Median [IQR]	31.3 (17.1–38.7]
Sex, % male	47.40%
Indication for HSCT	
CML	31.6% (6)
ALL	21.1% (4)
AML	15.8% (3)
Other	31.6% (6)
Stem cell donors	
Matched related	84.2% (16)
Matched unrelated	10.5% (2)
Haplo-identical	5.3% (1)
Myeloablative conditioning_1_	
TBI conditioning	73.3% (11)
Non-TBI conditioning	26.7% (4)
Presence of extra-pulmonary GVHD	
Skin	63.2% (12)
Eyes	47.4% (9)
Oral	47.4% (9)
Liver or GI tract	21.1% (4)
Time from HSCT to LTx	
Median [IQR]	10.2 [2.9–14.4]
Lung explant pathological diagnosis	
Bronchiolitis obliterans	78.9% (15)
Pleuro-parenchymal fibro-elastosis	21.1% (4)

^1^Conditioning regimen not known for four patients

The median time from HSCT to LTx was 10.2 [2.9–14.4 years] and the median LTx waiting list time was 2.1 [1.0–4.9] months. Explant pathology identified bronchiolitis obliterans in 15 of the allo-HSCT cohort (78.9%). In the propensity-score matched cohort, cystic fibrosis was the most common indication for LTx (60.5%, n = 23), followed by interstitial lung disease (28.9%, n = 11). Four patients in the allo-HSCT cohort (no patients in matched cohort) were receiving immunoglobin replacement for hypogammaglobulinemia (IgG deficiency) prior to LTx (p = 0.007). Three of the allo-HSCT cohort were also IgA deficient prior to LTx, 1 of which was receiving immunoglobulin replacement therapy for co-existing IgG deficiency. There were no statistically significant differences in recipient age, waiting list time or priority [13], immunosuppressive risk, donor-recipient CMV status, or the proportion of patients bridged to LTx between the LTx cohorts (Table 2). Donor age, type, requirement for ex vivo lung perfusion (EVLP) assessment and ischaemic times were also similar between these cohorts (Additional file 1: Table S3). There was no statistically significant difference in the incidence of post-LTx infections based on pre-LTx GVHD treatment (p = 0.810).

**Table 2:** Recipient characteristics at the time of lung transplantation

	Allo-HSCT cohort (n = 19)	Matched controls (n = 38)	p value
Age, years			
Median [IQR]	39.2 [29.6–48.7]	35.3 [25.9–54.3]	0.906
Sex, % male	47.40%	63.20%	0.273
BMI	19.7 ± 3.2	22.0 ± 4.9	0.07
Time on waiting list, months			
Median [IQR]	2.1 [1.0–4.9]	1.8 [0.8–6.2]	0.488
Indication for LTx	LONIPCs of allo-HSCT—100%	CF—60.5% (23)	N/A
		ILD—28.9% (11)	
		PAH—5.3% (2)	
		COPD—5.3% (2)	
Waiting listing priority			
Status 1	21.1% (4)	13.2% (5)	0.463
Status 2	42.1% (8)	57.9% (22)	0.279
Status 3	36.8% (7)	29.0% (11)	0.56
Mechanical or ECLS bridging to LTx	26.3% (5)	10.5% (4)	0.143
Double lung transplant	100%	100%	1
Donor-recipient crossmatch_1_			
Standard IS risk	89.5% (17)	94.7% (36)	0.855
Increased IS risk	10.5% (2)	5.3% (2)	
CMV status			
D−ve/R−ve	36.8% (7)	31.6% (12)	0.769
D−ve/R+ve	31.6% (6)	34.2% (13)	> 0.99
D+ve/R−ve	15.8% (3)	21.1% (8)	0.735
D+ve/R+ve	15.8% (3)	13.2% (5)	> 0.99
Hypogammaglobulinemia			
Pre LTx IgG level, g/L	11.2 ± 4.9	Not assessed	N/A
Pre LTx IVIg replacement	21.1% (4)	0%	0.007
Recipient co-morbidities			
Ex-smoker	26.3% (5)	26.3% (10)	> 0.99
Diabetes	21.1% (4)	29.0% (11)	0.751
GERD	10.5% (2)	34.5% (20)	0.077
Hypertension	15.8% (3)	13.2% (5)	> 0.99
Pre-LTx respiratory pathogen history			
Aspergillus	42.1% (8)	34.2% (13)	0.575
Pseudomonas	31.6% (6)	50% (19)	0.26
NTM	26.3% (5)	7.9% (3)	0.102
Burkholderia_2_	0% (0)	7.9% (3)	0.544
Recipient BAL at LTx			
Category 1 pathogens	26.3% (5)	36.8% (14)	0.555
Category 2 pathogens	5.3% (1)	18.4% (7)	0.247
Category 1 or 2	31.6%% (6)	39.5% (15)	0.772

^1^High IS risk is defined as a positive virtual or flow crossmatch at the time of transplant (and received antibody desensitisation at the time of transplant)

^2^Presence of *Burkholderia cepacia* species, but not *Burkholderia cenocepacia*

### Early post-transplant outcomes

Recipients of LTx from the allo-HSCT cohort spent 4.0 [2.0–25.5] days in ICU compared to the 3.3 [2.0–5.3] days of the control group (p = 0.298). The overall post-LTx hospital length of stay (LOS) for allo-HSCT cohort was 23.1 [17.0–101.0] days, compared to 17.0 [13.8–26.0] days in the control cohort (p = 0.009; Table 3). Survival to 90-days post-LTx was 100% in both cohorts.

**Table 3:** Post-lung transplant outcomes

	Allo-HSCT cohort (n = 19)	Matched controls (n = 38)	p value
ICU length of stay (LOS), days			
Median [IQR]	4.0 [2.0–25.5]	3.3 [2.0–5.3]	0.298
Post-LTx hospital LOS, days			
Median [IQR]	23.1 (17.0–101.0)	17.0 [13.8–26.0]	0.009
Biopsy proven acute cellular rejection_1_			
Any ACR grade	47.3% (9)	65.8% (25)	0.253
ACR > A1B0	21.1% (4)	34.2% (13)	0.370
ACR treatment in the 1st post-LTx year	31.6% (6)	52.6% (20)	0.165
Antibody mediated rejection			
Presence of dnDSA_2_	20% (4 of 15)	59% (22 of 37)	0.041
Time to DSA	73.5 days	87.0 days	0.777
Treatment for AMR	0%	21.1% (8)	0.042
CMV viraemia			
CMV D + /R- match	100% (3)	75% (6)	> 0.99
CMV D-/R + or D + /R + match	30% (3)	27.8% (5)	
BAL microbiology_3_			
Category 1 pathogens	63.2% (12)	76.3% (29)	0.356
Category 2 pathogens	57.9% (11)	36.8% (14)	0.163
Category 1 or 2	79.0% (15)	76.3% (29)	> 0.99
Severe infections_4_			
Percentage of patients	68.4% (13)	55.3% (21)	0.401
Incidence of severe infections	0.67 severe infections per patient per year	0.36 severe infections per patient per year	0.070_5_
Chronic rejection			
CLAD-free at 3 years	78.0%	74.9%	0.721
Median time to CLAD	4.98 years	Undefined	0.622
Graft survival			
1-year survival	100%	97.1%	0.473
5-year survival	50.0%	68.5%	0.161
10-year survival	25.0%	56.1%	0.059
CLAD-free survival			
3-year CLAD free survival	52.1%	71.1%	0.306
Median CLAD free survival	4.26 years	Undefined	0.142
Re-Transplant	0%	7.9% (3)	0.544

^1^TBBx performed within the first 12 months following lung transplant

^2^Presence of dnDSA during the 1st year post LTx

^3^BAL performed in the 1st 24 months following LTx. Category 1 and 2 pathogens classified as clinically significant and usually clinically significant, respectively

^4^Requiring in-patient admission or home IV treatment arranged. Excluded from this analysis were peri-operative infections (i.e., occurring during admission following transplant surgery) and infections that were managed as an out-patient with PO medication alone (e.g., CMV viraemia, NTM and Aspergillosis)

^5^This p value was derived from comparing hazard ratio with the Andersen-Grill model

### Acute rejection

**Fig.1:**
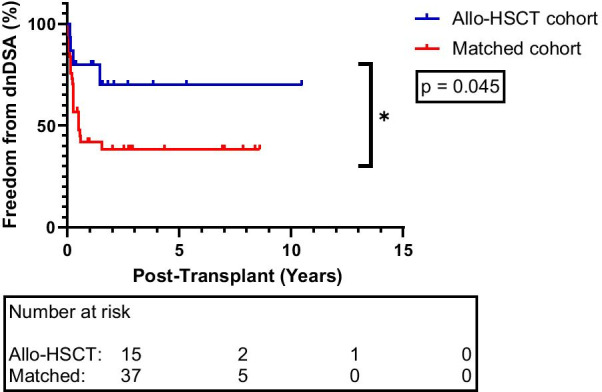
Freedom from de novo DSA following DLTx for LONIPCs of allo-HSCT compared to propensity-score matched controls

In the first post-LTx year, at least 1 episode of biopsy proven acute cellular rejection (ACR) of any grade was identified in 47.3% of the allo-HSCT cohort compared to 65.8% of controls (p = 0.253). During this period, 31.6% of the allo-HSCT cohort were treated for ACR compared to 52.6% of controls (p = 0.165) (Table 3). At 1-year post-LTx, 80% of the allo-HSCT cohort had remained free from dnDSA, compared to 41% of the controls (p = 0.041, Table 3). Overall, the allo-HSCT cohort experienced greater freedom from dnDSA (p = 0.045; Fig. 1). No patient in the allo-HSCT cohort received treatment for antibody mediated rejection (AMR), compared with 21.1% in the control cohort (p = 0.042; Table 3).

### Infection

Based on pre-LTx microbiology history, there was no statistical difference in the proportion of patients with a history of respiratory colonisation or infection due to *Aspergillus* species, *Pseudomonas Aeruginosa* or non-tuberculous mycobacteria. Airway flora at the time of LTx, assessed by recipient BAL, showed no difference in the proportion of patients colonised with category 1 (26.3% vs. 36.8%; p = 0.555) or category 2 (5.3% vs. 18.4%; p = 0.247) pathogens (Table 2).

In the 2 years following LTx (excluding peri-operative BAL results), there was no significant difference in the proportion of patients with at least one BAL culture that was positive for category 1 (63.2% vs. 76.3%; p = 0.356) or category 2 (57.9% vs. 36.8%; p = 0.163) pathogens (Table 3). Of the category 2 pathogen results, 52.6% of the allo-HSCT cohort culturing either *Aspergillus Fumigatus* or *Mycobacterium Avium*, compared to 29.0% of the matched cohort (p = 0.092).

Viral respiratory infections were identified (BAL or nasopharyngeal swab assessment) in 4 of the allo-HSCT cohort and 7 of the control cohort during follow up (21.1% vs. 18.4%; p > 0.999). This included 5 separate infections in the allo-HSCT cohort (Influenza × 3, RSV × 1, Coronavirus × 1) and 8 separate infections in the control (RSV × 5, Influenza × 3). RSV was managed as an inpatient with 5 days of inhaled ribavirin. Influenza cases were managed with oral oseltamivir as an outpatient.

The allo-HSCT cohort experienced 0.67 severe infections per year following LTx, classified as requiring either IV therapy or hospital admission (compared to 0.36 infections/patient/year in the control cohort). This corresponded to a hazard ratio for severe infection of 1.79 (95% CI 0.95–3.35; p = 0.07). CMV viraemia occurred in a similar proportion of patients in each cohort (Table 3). However, each LTx recipients with a primary CMV mismatch in the allo-HSCT cohort (CMV IgG D + /R-) required at least 1 admission for IV Ganciclovir (five episodes in three patients), with CMV disease being the cause of death in 1 case. In the matched cohort, CMV viraemia occurred in 6 of 8 CMV D + /R- LTx recipients, but only one required inpatient therapy (1 episode in 8 patients) and this was not associated with death. To assess the risk of severe CMV infections (i.e., requiring IV therapy) in LTx recipients with a primary CMV mismatch, we used a generalized estimating equations model adjusted for time to infection. The odds ratio for severe CMV infection was 8.94 with allo-HSCT (p = 0.056). Infection was the cause of death of 5 LTx recipients in the allo-HSCT cohort (CMV disease × 1, pneumonia × 2 and GI infection × 2) compared to 1 LTx recipient (bacteraemia × 1) in the propensity-score matched cohort. Using Gray’s K sample test, to compare the cumulative incidence of competing risk, there was an increased risk of death due to infection in the allo-HSCT cohort (Fig. 3a; p = 0.003).

### Chronic rejection, hematologic relapse and post-LTx survival

**Fig. 2:**
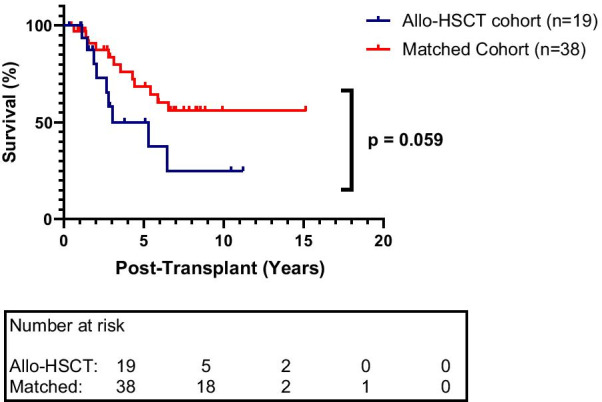
Graft survival following DLTx for LONIPCs of allo-HSCT compared to propensity-score matched controls

**Fig. 3:**
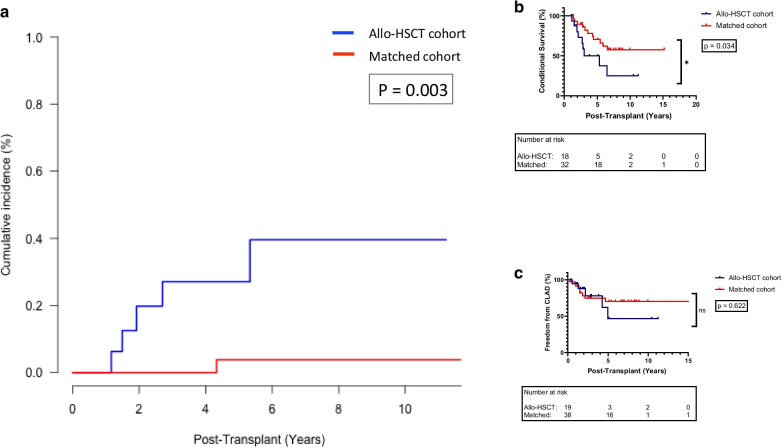
Comparison of outcomes following DLTx for LONIPCs of allo-HSCT compared to propensity-score matched controls: **a** Cumulative incidence of death due to infection; **b** Graft survival, conditional on 1-year survival; **c** Time to the development of CLAD following DLTx for LONIPCs of allo-HSCT compared to propensity-score matched controls

There was no statistically significant difference in overall graft survival (Fig. 2; p = 0.059) or time to CLAD onset (Fig. 3c; p = 0.622) between the allo-HSCT cohort to the propensity-score matched cohort. However, in analysis conditional on 1-year survival, graft survival was reduced in the allo-HSCT cohort (Fig. 3b, p = 0.034), equating to a hazard ratio for death of 3.04 (1.09–8.49). Within the allo-HSCT cohort, there was no significant difference in post-LTx survival based on pre-LTx diagnosis (p = 0.368), pre-lung transplant treatment for GVHD (prednisone vs. prednisone + other immunosuppression; p = 0.221), time from allo-HSCT to LONIPC (< 5 years vs. ≥ 5 years; p = 0.348), or explant pathology (obliterative bronchiolitis vs. PPFE; p = 0.160).

CLAD was the cause of graft failure or death in 4 (21.1%) of the allo-HSCT cohort and 9 (23.78%) of the matched cohort (p > 0.999). Three patients in the matched cohort underwent re-LTx for CLAD (Table 3). One patient developed a relapse of their underlying hematological malignancy (CML) post-LTx that was managed with Imatinib and is currently in complete molecular remission.

### Comparison to patients undergoing DLTx for CLAD

**Fig. 4:**
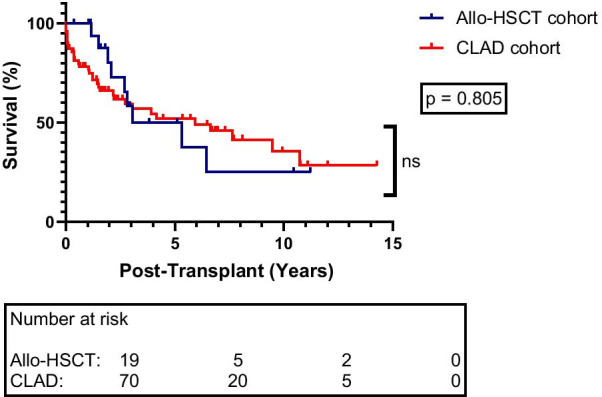
Survival following DLTx for LONIPCs of allo-HSCT compared to DLTx for CLAD (1st re-transplant), between 2003 and 2019

The observations that antibody-mediated rejection was less frequent, and infection was more frequent or severe, suggested a higher degree of immunosuppression in patients with allo-HSCT compared to propensity-matched LTx controls. Since patients requiring a re-transplant (re-LTx) for CLAD have a history of chronic immunosuppression, we elected to compare outcomes of patients undergoing a first re-LTx (DLTx only; n = 70; Additional file 1: Table S4) to that of the allo-HSCT cohort, within the same study period. Biopsy proven ACR (TBBx > A1B0) was identified in 10% of the re-LTx cohort, compared to 21.1% in the allo-HSCT cohort (p = 0.239). Overall post-LTx graft survival was similar (Fig. 4; p = 0.805) between cohorts, but there was a time-dependent mortality risk that differed between each cohort. The CLAD cohort had a higher risk of death during the early post-LTx phase (100% vs. 78.6% 1-year survival; p = 0.034). However, in patients surviving 1-year post-LTx, the allo-HSCT cohort experienced a hazard ratio of 1.99 for death (95% CI 0.79–5.01; p = 0.145) and 1.59 for infection-related death (95% CI 0.43–5.95; p = 0.489).

## Discussion

In this study, we report that patients who undergo LTx for LONIPCs of allo-HSCT have an estimated 5-year survival of 50%. This compares favourably to the reported outcomes of patients who develop LONIPCs but do not undergo LTx [5, 14]. However, in analysis conditional on 1-year survival, the allo-HSCT cohort experienced an increased mortality risk compared to propensity-score matched controls. The time to CLAD onset was similar in each cohort, but the allo-HSCT cohort experienced an increased risk of death due to infection. These results, added to lower rates of AMR, suggest that allo-HSCT LTx recipients experience a relatively increased state of immunosuppression compared to other LTx recipients.

Comparing outcomes of LTx for LONIPCs of allo-HSCT to outcomes of re-LTx for CLAD (within the same era), survival during the first post-LTx year was similar. However, following this 1st post-LTx year, there was a non-significant trend towards infection related deaths in the allo-HSCT cohort that we hypothesise may be the result of  acquired immunodeficiencies related to allo-HSCT and not present in the re-LTx cohort.

Infectious complications occurring after allo-HSCT have previously been well described, with CMV infection and invasive aspergillosis being noteworthy [15–17]. The potential for serious infection in LTx recipients with a history of allo-HSCT has also been reported [18–20]. In the largest study of LTx for this indication, infection accounted for 41% of deaths during follow-up [21]. The authors identified the timing of allo-HSCT (within 2 years) and bridging to LTx (mechanical ventilation or ECLS) as risk factors for early sepsis related-mortality [21]. In our study, 26.5% of patients were bridged to LTx and 2 patients underwent LTx within 2 years of allo-HSCT, however survival to 1-year was 100%. Whether the improvement in early outcomes seen in this study is due to lack of statistical power or driven by differences in peri-operative immunosuppressive or anti-microbial therapy is an important question that should be answered by a prospective trial.

In attempting to explain the increased infection related mortality seen in this study, we hypothesize that acquired immunodeficiencies related to allo-HSCT alter the risk profile of these patients. We further hypothesize that the persistence of these acquired immunodeficiencies is the  result of impaired or delayed immune reconstitution following allo-HSCT. Whilst functional innate immunity is generally thought to  recover within a few months of allo-HSCT, a significant proportion of patients  experience adaptive immune deficits at 1-year [22] and ongoing immunological deficits present at greater than 10 years have been reported [23]. Unfortunately, detailed assessment of the pathways underpinning prolonged immunodeficiency are lacking, although T- and B- cell memory responses, T-cell diversity and regulatory T-cell balance have been implicated [24]. This immunological dysfunction is likely further exacerbated by chronic GVHD [25], which may further delay T-cell immune reconstitution [26] and potentiate B-cell deficiency [27]. It is notable that all patients in this study experienced chronic GVHD affecting at least 1 organ at the time of LTx.

The optimal approach for translating knowledge regarding potential immunodeficiency and resultant infection risk into post-LTx management is not clear cut. It might be argued that a less intensive immunosuppressive regimen should be provided to LTx recipients with previous allo-HSCT. However, the complexity of the pathobiology and the impact of individualised factors in the pathogenesis of these deficits, makes a “one-size-fits-all” strategy inappropriate for this cohort. Our study suggests that these patients commonly experience humoral immune deficiencies, evidenced by the high proportion of patients receiving immunoglobulin replacement and the low rates of DSA development post-LTx. However, T-cell mediated rejection (ACR) remains an ongoing concern, with 31.6% of the allo-HSCT cohort requiring treatment for this during the first post-LTx year—similar to ACR rates reported by international registries (for all LTx recipients) [28]. Furthermore, when compared to re-LTx for CLAD, a greater proportion of the allo-HSCT cohort developed mild-moderate ACR (21.1% vs. 10.0%; p = 0.239).

Balancing the impact of acquired immunodeficiencies on graft function, infection and rejection in this situation is therefore quite complex. We would consider the most prudent approach to be careful assessment of the degree of impairment of host immunity prior to LTx, followed by a personalised immunosuppression strategy post-LTx with careful monitoring. In moving the field towards precision medicine, the development of assays that allow for personalised immunosuppressive approaches remains a key research priority [29]—as highlighted by this study. However, while we wait for this breakthrough, strategies to optimise post-LTx outcomes in allo-HSCT recipients might include prolonged CMV prophylaxis [30], pre-emptive anti-microbial therapy [31], monitoring for immunoglobulin deficiency, and in appropriate cases, careful reduction of maintenance immunosuppression (compared to standard regimens).

The conclusions drawn from this study are limited by retrospective design, sample size and inclusion period. However, this study adds to the limited evidence base regarding post-LTx outcomes for this rare indication. Furthermore, ongoing increases in allo-HSCT activity may lead to LONIPCs becoming a more frequent indication for LTx in the future. As such, improving our understanding of potentially modifiable risk factors is important if outcomes are to improve. To summarise, LTx is a feasible option for patients with LONIPCs of allo-HSCT but post-LTx infectious complications are a concern and evidence-based strategies to reduce this risk are required.

## Supplementary Information

**Additional file 1: Table S1**. Classification of BAL pathogens. **Table S2**. Classification of waiting list status^2^. **Table S3**. Lung donor characteristics. **Table S4**. Recipient characteristics at the time of re-transplant for chronic lung allograft dysfunction.

## Data Availability

The datasets during and/or analysed during the current study available from the corresponding author on reasonable request.

## Abbreviations

ACR: Acute cellular rejection
allo-HSCT: Allogeneic hematopoietic stem cell transplantation
CXM: Antibody cross match
AMR: Antibody mediated rejection
ATG: Anti-thymocyte globulin
BAL: Bronchoalveolar lavage
cGVHD: Chronic graft-versus-host-disease
CLAD: Chronic lung allograft dysfunction
CML: Chronic myeloid leukaemia
CMV: Cytomegalovirus
DLTx: Double lung transplantation
DSA: Donor specific antibody
EVLP: Ex vivo lung perfusion
ECLS: Extracorporeal life support
LONIPCs: Late-onset non-infectious pulmonary complications
LOS: Length of stay
LTx: Lung transplantation
PCR: Polymerase-chain reaction
re-LTx: Re-transplantation
TBI: Total body irradiation
TBBx: Transbronchial lung biopsy
