# Supplementary material for: Lung transplantation for late-onset non-infectious chronic pulmonary complications of allogenic hematopoietic stem cell transplant
Source: Respir Res. 2021 Apr 7;22:101. doi: 10.1186/s12931-021-01699-8 (PMC8025894; doi:10.1186/s12931-021-01699-8)
Supplement: Supplementary file 1 — Additional file 1: Table S1. Classification of BAL pathogens. Table S2. Classification of waiting list status2. Table S3. Lung donor characteristics. Table S4. Recipient characteristics at the time of re-transplant for chronic lung allograft dysfunction. [file 12931_2021_1699_MOESM1_ESM.docx]

**Additional file**

**Table S1 Classification of BAL pathogens**

| Category | Pathogen classification | Examples of pathogens within each category |
| --- | --- | --- |
| 1 | Significant pathogens | Staphyloccocus aureus; Pseudomonas aeruginosa; Influenza A; Mycobacterium abscessus; Pneumocystis jiroveci. |
| 2 | Usually significant pathogens | Aspergillus fumigatus/niger/flavus; Mycobacterium Avium; Bordetella species; Scedosporium species. |
| 3 | Usually insignificant pathogens | Bordetella bronchiseptica; Enterovirus; Ralstonia picketti; Scopulariopsis species; Trichoderma species. |
| 4 | Insignificant pathogens | Penicillium species; Claposporium species; Phialophora species; Paecilomyces species. |
| 5 | Negative |  |
| 6 | Other |  |
| 7 | Missing data |  |

This pathogen classification system was designed by independent review from two experienced transplant infectious disease experts (Dr S Husain and Dr C Rotstein) in consultation with the lung transplant team^1^.

**Table S2 Classification of waiting list status**^2^

| Status | Medical Criteria |
| --- | --- |
| 3 | Rapidly deteriorating |
| 2 | Decompensation |
| 1 | Out of hospital (stable and waiting) |

**Table S3 Lung donor characteristics.**

|  | Allo-HSCT cohort (n=19) | Matched controls (n=38) | P value |
| --- | --- | --- | --- |
| Donor age, years | 47.5 ± 17.6 | 39.8 ± 17.9 | 0.130 |
| Donor-recipient sex matching  Matched  Unmatched | 78.9% (15)  21.1% (4) | 86.8% (33)  13.2% (5) | 0.463 |
| Donor type, % DBD_1_ | 84.2% (16) | 78.9% (30) | 0.906 |
| Donor EVLP assessment | 15.8% (3) | 21.1% (8) | 0.906 |
| Ischaemic time_2_  Warm ischaemic time  Total ischaemic time | 64.6 ± 15.0  444 ± 168 | 62.9 ± 18.7  527 ± 226 | 0.750  0.264 |

**1.** DBD - Donation after brain death **2.** Average of left and right ischaemic times.

**Table S4 Recipient characteristics at the time of re-transplant for chronic lung allograft dysfunction**

|  | CLAD cohort (n=70) |
| --- | --- |
| Age, years  Median (range) | 34.4 (19.7-41.4) |
| Sex, % male | 45.7% |
| BMI | 19.6 ± 4.9 |
| Time on waiting list, months  Median (range) | 3.9 (0.0-30.4) |
| Indication for LTx |  |
| Waiting listing priority  Status 1  Status 2  Status 3 | 10% (7)  32.9% (23)  57.1% (40) |
| Mechanical or ECLS bridging to LTx | 11.4% (8) |
| Double lung transplant | 100% |
| Donor-recipient crossmatch  Standard IS risk  Increased IS risk* | 84.3% (59)  15.7% (11) |
| CMV status  D-ve/R-ve  D-ve/R+ve  D+ve/R-ve  D+ve/R+ve | 17.1% (12)  27.1% (19)  7.1% (5)  48.6% (34) |

**References**

1. Levy L, Huszti E, Tikkanen J, et al. The impact of first untreated subclinical minimal acute rejection on risk for chronic lung allograft dysfunction or death after lung transplantation. *Am J Transplant* 2020; **20**(1): 241-9.

2. Wait list, organ offers and allocation policies. 2019. https://www.giftoflife.on.ca/resources/pdf/healthcare/TP_9_100_Dec1719.pdf.
